# Background Concentrations of Cultivable, Mesophilic Bacteria and Dust Particles in the Air in Urban, Rural and Mountain Regions

**DOI:** 10.3390/ijerph17249572

**Published:** 2020-12-21

**Authors:** Doris Haas, Angela Kriso, Theresa Fritz, Herbert Galler, Juliana Habib, Mihaela Ilieva, Michael Kropsch, Petra Ofner-Kopeinig, Martin Stonitsch, Andreas Strasser, Eduard Zentner, Franz F. Reinthaler

**Affiliations:** 1 D&R Institute of Hygiene, Microbiology and Environmental Medicine, Medical University of Graz, 8010 Graz, Austria; krisoangela@gmail.com (A.K.); theresa.fritz@medunigraz.at (T.F.); he.galler@medunigraz.at (H.G.); juliana.habib@medunigraz.at (J.H.); mihaela.ilieva@edu.uni-graz.at (M.I.); martin.stonitsch@medunigraz.at (M.S.); andreas.strasser@medunigraz.at (A.S.); franz.reinthaler@medunigraz.at (F.F.R.); 2 Agricultural Research and Education Center Raumberg Gumpenstein, 8952 Irdning, Austria; michael.kropsch@raumberg-gumpenstein.at (M.K.); eduard.zentner@raumberg-gumpenstein.at (E.Z.); 3 Institute for Medical Informatics Statistics and Documentation, Medical University of Graz, 8036 Graz, Austria; petra.ofner@medunigraz.at

**Keywords:** background concentrations, dust particles, mesophilic bacteria, meteorological factors, *Staphylococcus* sp., *S. aureus*

## Abstract

Particulate air components can be of anthropogenic or natural origin. It is assumed that in different geographical areas varying concentrations of mesophilic bacteria are present in the ambient air. The aim of this study was to determine the background concentrations of airborne culturable mesophilic bacteria and particulate matter in the ambient air. Furthermore, the association between their concentrations and some environmental factors was analysed. In the period from July to October 2019, concentrations of mesophilic bacteria and dust particles were measured in urban, rural and mountain areas using the single-stage air sampler and the particle counter. The concentrations of bacteria and dust particles in the air were counted as number of Colony Forming Units per cubic metre (CFU/m^3^) and particles per cubic metre (pa/m^3^). *Staphylococcus* sp. were identified. The median values of the cultivated mesophilic bacteria at 30 °C and 37 °C were 7.1 × 10^2^ CFU/m^3^ and 2.3 × 10^1^ CFU/m^3^ in mountain regions, 1.3 × 10^2^ CFU/m^3^ and 6.9 × 10^1^ CFU/m^3^ in rural regions and 2.1 × 10^2^ CFU/m^3^ and 6.5 × 10^1^ CFU/m^3^ in urban regions. The median of *Staphylococcus* sp. was 2.5 × 10^0^ CFU/m^3^ in alpine areas and 7.5 × 10^0^ CFU/m^3^ in urban and rural areas. Higher bacterial concentrations were measured in sunshine and in windy weather. A relationship was observed between the concentrations of airborne mesophilic bacteria and the coarse particles in all three areas. The present study determined values between 5.0 × 10^0^ and 4.6 × 10^2^ CFU/m^3^ as natural background concentrations of airborne mesophilic bacteria and 1.2 × 10^7^ pa/m^3^ and 6.5 × 10^4^ pa/m^3^ for fine and coarse particles, respectively. These results can be proposed as baseline for the assessment of the emission sources of mesophilic bacteria for summer and early autumn.

## 1. Introduction

The concentration of aerosol particles in the environment is subject to considerable fluctuation. Dust particles can absorb significant amounts of microorganisms from the air. The concentration of airborne microorganisms depends essentially on factors such as vegetation, weather, time of year and day, traffic volume, environment, as well as the meteorological and climatic situation [1].

Basically, the concentrations of airborne mesophilic bacteria are the highest in the immediate vicinity of their sources, but particles can travel long distances due to wind and thermals [2]. Most biological particles are between 1 and 10 µm in size [3]. Natural background concentrations of atmospheric dust and bioaerosols are found globally but vary from region to region. Santl-Temkiv et al., 2020 conducted studies dealing with bioaerosol measurements from different regions worldwide [4]. The particulate aerosols contain about 25% bioaerosols. In some geographic regions bioaerosols can even reach up to 75% [5]. Particles which are emitted by both anthropogenic and natural sources vary greatly in concentration and can increase greatly in ambient air [6,7]. As it cannot be avoided that humans and animals are exposed to air particles, even only those emitted by natural sources, this research area is of interest to a broad target group.

Dust particles and bioaerosols in the ambient air interact constantly. It is assumed that the microbial load in the ambient air depends on the size of the suspended dust particles [8]. The size fractions of the suspended particles are mostly recorded in Particulate Matter (PM), which is based on the “National Air Quality “standard for particulate matter (“PM-Standard”) of the USA. A distinction is made between coarse particles and fine particles [9]. The size of the coarse particles ranges from >2.5 to 10 µm and of the fine particles from 0.1 to 2.5 µm [10]. The airborne microorganisms are often bound to solid particles with an aerodynamic diameter of more than 5.0 μm. The smaller particle fractions <5.0 μm tend to be practically free of microorganisms [11]. It is assumed that bacteria that are bound to larger particles retain their cultivability because the particles protect them from environmental stress [8]. Bioaerosols fluctuate in their concentrations during the course of the day and the year and are also exposed to different stress factors (e.g., dehydration or radiation), which can affect their viability [12,13,14].

Initially, it was assumed that microorganisms in the air are inactive or even no longer viable due to external circumstances that limit their survival [15]. However, mesophilic bacteria are very adaptable microorganisms and can therefore be found in a wide variety of habitats [16]. The viability of microorganisms is fundamentally dependent on the size of aerosol particles, air pollutants, sunlight, ambient temperature and the relative humidity of the environment [17]. The latest findings show that microbes can reproduce and metabolize in cloud droplets [18]. There are species that can survive longer in dry air, e.g., *Staphylococcus* and *Streptococcus*. *Staphylococcus aureus* can be found in dust or dry material in viable condition after months [19,20]. According to Morris et al., 2008, Gram-positive and spore-forming bacteria such as *Bacillus* sp. in the atmospheric air are more resistant to dehydration than Gram-negative bacteria because they have different cell wall structures [21]. This is a reason why Gram-positive microorganisms, especially bacilli and staphylococci, are more present in summer with higher coarse particle loads [22].

Little is known about the background concentrations of cultivable mesophilic bacteria and dust particles in the air, but these background concentrations are often used as reference values. Therefore, it is necessary to carry out studies in different geographical regions concerning potential sources of airborne mesophilic bacteria and how emissions, climatological and meteorological factors and other influences can affect bacterial communities in the air.

The aim of this study was to investigate the concentrations of cultivable mesophilic bacteria and dust particles in the ambient air in urban, rural and mountain regions. The main focus was on the presence of *Staphylococcus* sp. The influence of environmental factors on the mesophilic bacteria in the ambient air was examined by recording altitude and weather conditions such as temperature, humidity, wind, cloud cover and sunshine. The data of natural background concentrations of mesophilic bacteria and *Staphylococcus* sp. of this study can be used as baseline values for the assessment of emission sources.

## 2. Materials and Methods

### 2.1. Sampling Sites

Measurements of airborne mesophilic bacteria and particles were carried out at 25 different geographical locations in the Austrian province of Styria from July to October 2019. With the cooperation of the Provincial Government of Styria the locations and parameters were chosen according to the existing meteorological measuring stations. All background measuring points were located in areas with low or no emission sources and immissions.

Nine measuring locations were selected in “urban” regions having more than 8000 inhabitants, a high density of buildings and a high volume of traffic. Eight measurement sites with a population of less than 5000 inhabitants, low building density and traffic volume as well as a low industrial density were assigned as “rural” regions and eight measurement sites in “mountain” regions.

The above criteria of urban, rural and mountainous regions are defined by Statistic Austria. https://www.statistik.at/web_en/classifications/regional_breakdown/urban_rural/index.html. The measuring locations were selected in three different altitudes level: (a) 250 m to 450 m; (b) 450 m to 850 m and (c) above 850 m above sea level.

**Figure 1:**
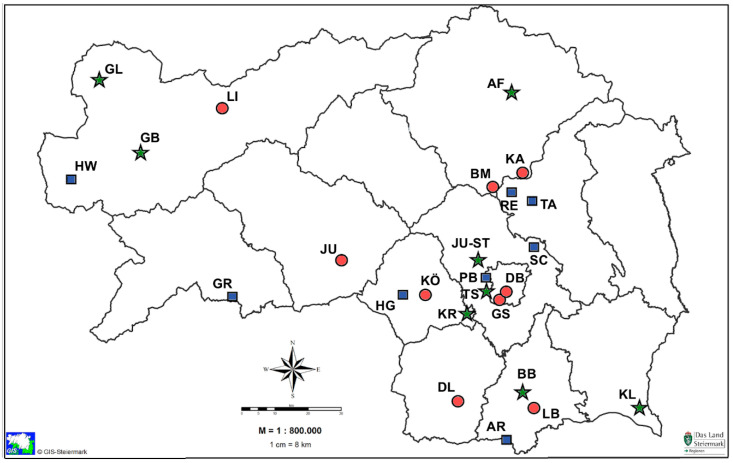
Map of Styria, Austria with the measuring locations [23] (Edited by A. Kriso). The sym-bols urban “
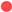
”; rural “
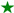
”; mountain “
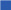
” were used in reference to the sampling location.

The measurements (*n* = 500) were carried out in the 25 locations on five different days and four air samples were collected at each site. The measurements were conducted in three week intervals between 10:00 a.m. and 2:00 p.m. The influence of different temperature or humidity levels, wind force and the intensity and effects of sunshine or cloud cover on the airborne mesophilic bacterial concentration measurements were observed. The sampling sites were located as far as possible from obvious emission sources (Figure 1).

### 2.2. Measuring Devices and Processing of the Samples

The microbial air sampler MAS-100NT^®^ (MBV AG, Stafa, Switzerland) was used to capture bioaerosols from the air for two minutes at a flow rate of 100 L/min. The optical particle counter OPC-N3 (Alphasense, London, UK) was used for particle concentration measurements. This device detects particles from 0.35 to 40 μm and assigns them to one of 24 size channels [24,25]. A sensor for temperature and relative humidity was used for recording the meteorological parameters (Testo GmbH, Wien, Austria). The wind velocity was taken from online data of the Provincial Government of Styria [23]. Wind velocity in metres per second (m/s) was categorised into: windless: 0–3 m/s; slight wind: >3–5 m/s and strong wind: >5 m/s.

The culture media Tryptic Soy Agar (TSA) with cycloheximide and Mannitol Salt Agar (MAN) were used in duplicate for the collection of air samples. TSA was used to determine the total concentrations of mesophilic bacteria and MAN for staphylococci. The cultivation was done at 30 °C and 37 °C for total mesophilic bacteria and at 37 °C for staphylococci for 48 h, then the colonies were counted and converted into the number of colony-forming units per cubic metre of air (CFU/m^3^). Based on the colour reaction of the MAN media, yellow colonies were presumed to be *S. aureus*. To obtain pure cultures, these colonies were transferred to blood agar (24 h, 37 °C). Subsequently, the subcultures were qualitatively examined by means of VITEK MS (bioMérieux, France), a MALDI-TOF mass spectrometry system. All identifications displaying a single result with a confidence value of 99.9% were considered acceptable for VITEK MS. Isolates yielding a single or multiple result without acceptable confidence level as well as no identification results were retested [26,27]. The retesting of bacterial identification was done using polymerase chain reaction (PCR), amplified 16S rRNA gene sequence and comparing sequences with those available in the GenBank, EMBL and DJB databases using the gapped BLASTN 2.10.1 programme through the National Center for Biotechnology Information server [28,29].

### 2.3. Statistical Analysis

Statistical analyses were performed and charts generated by using SAS 9.4. All values are described with their median, minimum, maximum and interquartile range. To examine the relationship between particle and airborne mesophilic bacterial concentrations, the partial correlation coefficient was calculated and correlated with temperature and humidity. The particle concentrations were measured by OPC-N3 device and evaluated with MATLAB version R2019a software. The registered particles of all size channels (0.35–40 μm) were extrapolated to obtain pa/m^3^ of air. The particles were categorised as fine (0.35–2.3 µm) and coarse (>2.3–10 µm). Finally, the data of the fine and coarse particles (pa/m^3^) were compared with the mesophilic bacterial concentrations (CFU/m^3^).

## 3. Results

For the presentation of the study data in figures, outliers represented as dots, are cases with values between 1.5 and 3 times the interquartile range and extreme values represented as stars, are cases with values more than 3 times the interquartile range.

### 3.1. Background Concentrations of Mesophilic Bacteria and Dust Particles in the Styrian Province

The median of all measurement values in the province of Styria were calculated for the airborne mesophilic bacteria and particle concentrations. The total concentrations of airborne mesophilic bacteria, cultivated at 30 °C and 37 °C were 1.4 × 10^2^ CFU/m^3^ and 5.3 × 10^1^ CFU/m^3^, respectively. The median concentration of *Staphylococcus* sp. was 5.0 × 10^0^ CFU/m^3^. The background values for fine and coarse particles were 1.2 × 10^7^ pa/m^3^ and 6.5 × 10^4^ pa/m^3^, respectively.

### 3.2. Total Mesophilic Bacterial Concentrations

**Figure 2:**
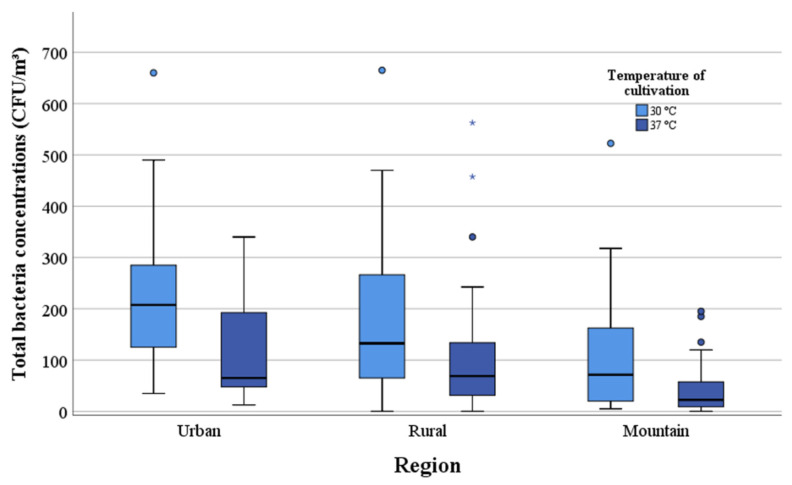
Comparison of the total mesophilic bacterial concentrations (CFU/m^3^) in mountain, rural and urban regions at 30 °C and 37 °C.

In the mountain regions, the median values of the total mesophilic bacterial concentrations for the eight measuring sites were 7.1 × 10^1^ CFU/m^3^ and 2.3 × 10^1^ CFU/m^3^ for the cultivation at 30 °C and 37 °C, respectively. The values in the mountain regions were below the median values obtained from the eight rural (1.3 × 10^2^ CFU/m^3^ and 6.9 × 10^1^ CFU/m^3^) and nine urban (2.1 × 10^2^ CFU/m^3^ and 6.5 × 10^1^) locations (Table 1). At 30 °C, the mesophilic bacterial concentrations in the rural and urban regions were twice and three times higher than in the mountain regions. The results of cultivation at 37 °C for the samples from rural and urban regions were three times higher than the median values of the mountain regions. The highest median of the three geographic areas was obtained in the urban regions. Figure 2 shows the total mesophilic bacterial concentrations at 30 °C and 37 °C for cultivation of the three regions.

The total mesophilic bacterial concentration cultivated at 30 °C was noticeably the highest in AR and HG among the mountainous locations. The rural locations BB, KL, JU-ST and TS also had high median concentrations in the three digits range. The highest concentrations of the total mesophilic bacteria were measured at 30 °C and 37 °C, at DB and GS in the urban region (Table S1).

### 3.3. Concentrations of Staphylococcus spp.

**Figure 3:**
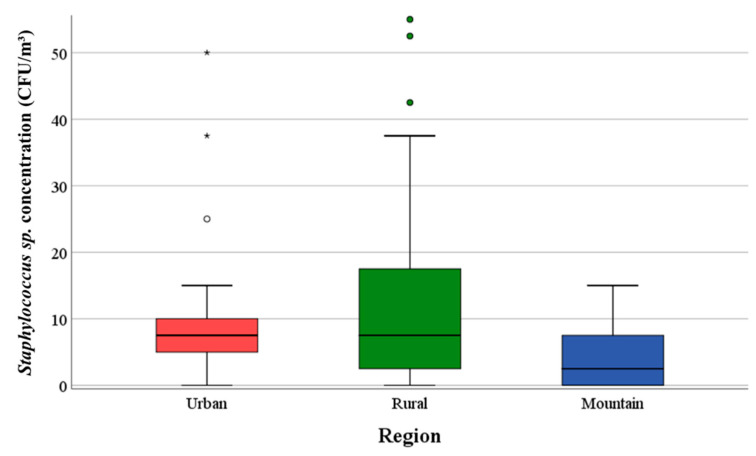
Comparison of the concentrations of *Staphylococcus* sp. of the three regions in CFU/m^3^.

The median value for concentrations of *Staphylococcus* sp. was 3.0 × 10^0^ CFU/m^3^ at the eight measuring sites in the mountains. This value is significantly (*p* < 0.001) lower than the values of the eight rural and nine urban locations. Figure 3 shows the *Staphylococcus* sp. concentrations of the three geographic regions.

Staphylococci were detected with a median value of 5.0 × 10^0^ CFU/m^3^ in the mountain regions up to 1200 m above sea level at the locations HG, PB, RE and TA. The rural locations BB and KL and the urban locations GS, DB, LB and LI had the highest median values of *Staphylococcus* sp. For example, at the urban site LB, the concentrations ranged between 7.5 × 10^0^ and 7.5 × 10^1^ CFU/m^3^ (Table S1).

In each geographical region the proportion of staphylococci was approximately 11% of the total mesophilic bacteria cultivated at 37 °C.

Pure cultures of 47 macroscopical suspected colonies for *S. aureus* were analysed, but no *S. aureus* colony could be identified.

### 3.4. Environmental Factors Influencing Mesophilic Bacterial Concentrations

**Figure 4:**
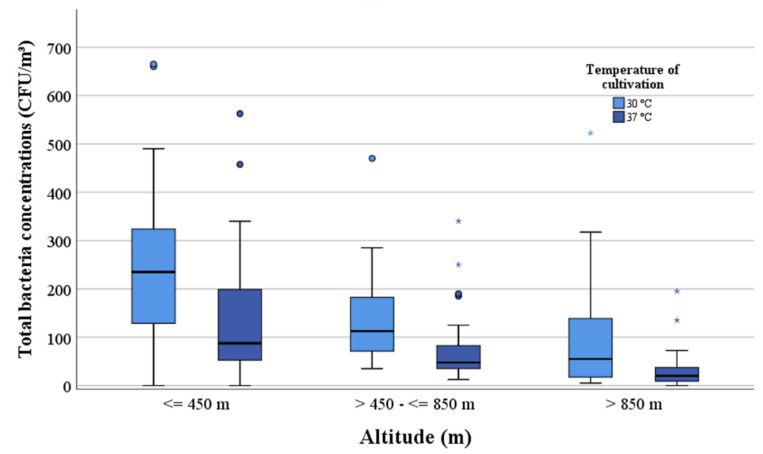
Comparison between the concentrations of the total mesophilic bacteria (CFU/m^3^) and altitude (m).

**Figure 5:**
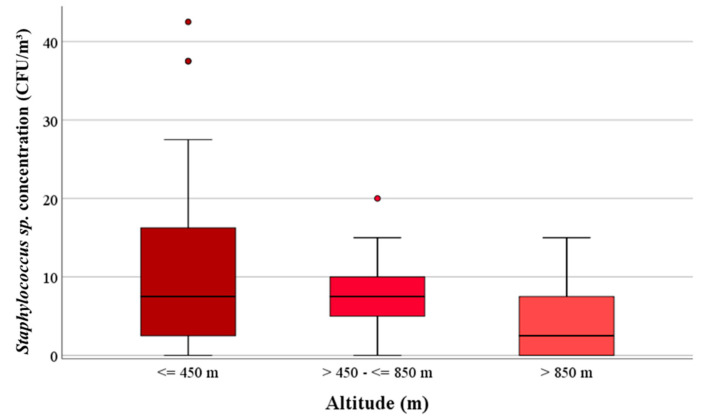
Comparison between the concentrations of *Staphylococcus* sp. (CFU/m^3^) and altitude (m).

The median values of the total mesophilic bacterial and *Staphylococcus* sp. concentrations are listed in Table 1 in relation to altitude and weather conditions. Higher median values of the total mesophilic bacteria and *Staphylococcus* sp. were found at an altitude below 850 m (Figure 4 and Figure 5). The concentrations of staphylococci decreased with increasing altitude and no staphylococci could be detected above an altitude of 1200 m. The correlations between the total mesophilic bacterial concentrations (30 °C) or the *Staphylococcus* sp. and altitude were negative (rho = −0.48 and −0.34).

The air humidity at all the measurement locations ranged between 25.7% and 99.0%, and the temperature between 0.5 °C and 35.7 °C. All mesophilic bacterial concentrations cultivated at 30 °C and 37 °C correlated with air humidity (rho = −0.35 and rho = −0.38) and temperature (rho = 0.48 and rho = 0.52). With increasing air humidity, mesophilic bacterial concentrations decreased. However, when the air temperature rose, mesophilic bacterial concentrations increased in the ambient air. The same results for correlation analysis were obtained for the concentrations of staphylococci.

According to other weather conditions, the concentrations of the total mesophilic bacteria cultivated at 30 °C and 37 °C were 1.3 and 1.5 times higher in sunshine than in heavily overcast weather. There was no discernible influence of sun on the concentrations of *Staphylococcus* sp., on the contrary, the concentrations were 1.6 times higher in sunshine than in heavily overcast weather. In foggy weather, the concentrations of the mesophilic bacteria in the air were always the lowest (<20 CFU/m^3^).

As mentioned above, wind velocity is divided into three categories: windless, slight wind and strong wind. The results of the total mesophilic bacterial concentrations at 30 °C were the highest in slight wind, lower in strong wind and lowest in windless weather. The concentrations of the total mesophilic bacteria cultivated at 37 °C were the highest when there was no wind, lower in slight wind and the lowest in strong wind. Concentrations of *Staphylococcus* sp. were the highest in slight wind but lowest in strong wind.

### 3.5. Concentrations of Dust Particles

**Figure 6:**
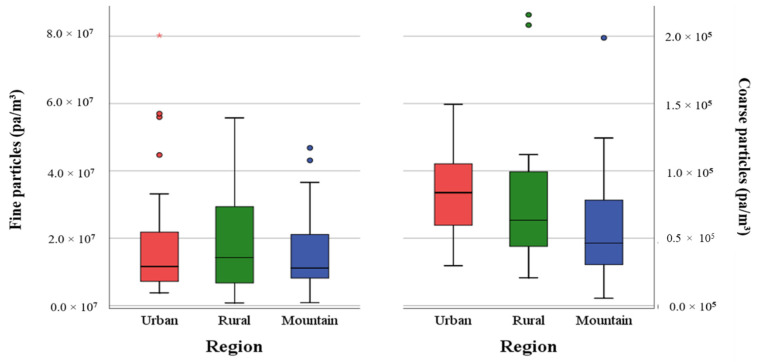
Comparison of fine and coarse particle concentrations pa/m^3^ in the three regions.

In the three investigated regions, the number of fine particles (0.35–2.3 μm) ranged from 7.98 × 10^5^ to 8.02 × 10^7^ pa/m^3^ and of coarse particles (>2.3–10 μm) from 5.48 × 10^3^ to 9.94 × 10^4^ pa/m^3^, respectively (Table 2). The fine particle numbers in the rural regions were 1.2 times higher than in the urban areas and 1.1 times higher than in the mountains. The highest concentrations of fine particles were found in the rural regions. The coarse particles showed differences in the concentrations between the urban, rural and mountain regions. While the median values in the rural regions were 1.4 times higher than those of the mountain regions, the median value in the urban area was twice as high as in the mountain regions. The lowest median value was found in the mountain regions (Figure 6).

### 3.6. Environmental Factors Influencing the Number of Dust Particles

**Figure 7:**
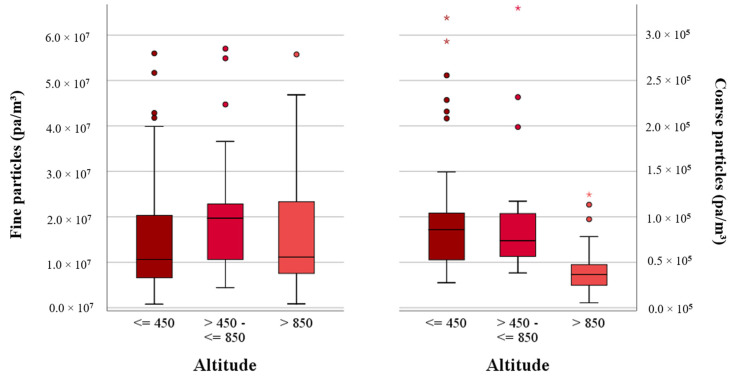
Correlation between fine and coarse particles (pa/m^3^) and altitude (m).

In Table 2, the median values of the dust particle numbers are shown in Table 2 in relation to altitude and weather conditions of sun, clouds and wind velocity. Higher median values of the fine particle concentrations were found between >450 m and ≤850 m altitude. The highest numbers of coarse particles were found in an altitude <450 m with a value of 8.58 × 10^4^ pa/m^3^. The lowest fine and coarse particle numbers were detected above an altitude of >850 m (Figure 7). No correlation was found between fine particles and altitude. The correlation between coarse particles and altitude was negative (rho = −0.39).

Air humidity and air temperature had an influence on the dust particles. The fine particles were affected positively by air humidity (rho = 0.28) and only slightly by air temperature (rho = 0.12). However, the coarse particle concentrations did not correlate with air humidity but were also slightly affected by air temperature (rho = 0.18).

The fine particles showed nearly the same median values in all weather conditions whereas the mesophilic bacterial counts in sunny weather were up to 1.3 times higher than in slightly overcast weather. In contrast to the fine particles, the coarse particles were not found in foggy weather. The results show that dust particles were present in the air in sunny weather.

Without wind the median concentrations of fine particles were twice as high as those in slight or strong wind conditions. The lowest numbers of fine particles were found in strong wind conditions. The coarse particle concentration was highest when there was either no wind or strong wind with more than 5 m/s. However, a slight wind velocity of 0–3 m/s reduced the number of coarse particles in the air.

### 3.7. Comparison of Mesophilic Bacterial and Particle Concentrations

**Figure 8:**
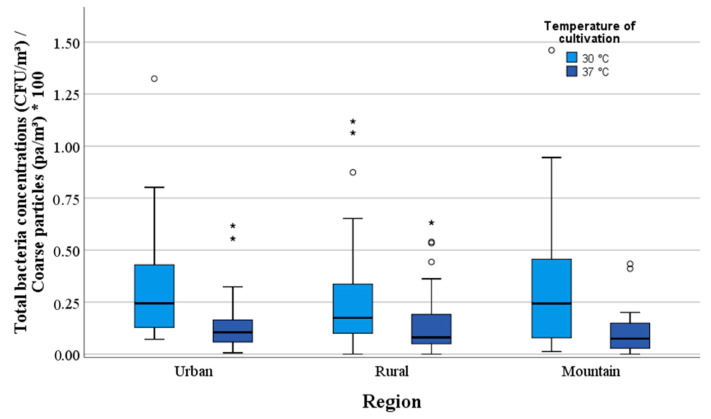
Proportion between coarse particles (pa/m^3^) and bacterial concentrations (CFU/m^3^) in the three regions.

**Figure 9:**
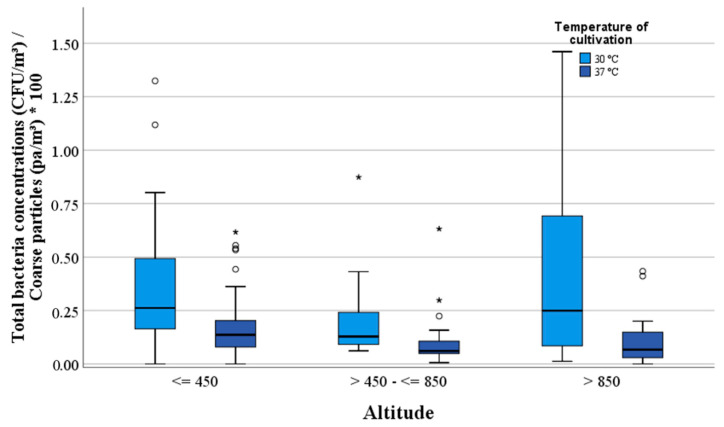
Proportion between coarse particles (pa/m^3^) and bacterial concentrations (CFU/m^3^) in different altitude (m).

Fine and coarse particles in pa/m^3^ were compared with the number of CFU/m^3^ of mesophilic bacteria in the ambient air. The present study did not find a correlation between mesophilic bacterial concentrations at 37 °C and fine particles numbers. There was only a slightly negative correlation between fine particles and the concentrations of mesophilic bacteria at 30 °C. The coarse particles showed a correlation to the mesophilic bacterial concentrations cultivated at 30 °C (rho = 0.39) and 37 °C (rho = 0.45). The proportion between the coarse particles per cubic metre air (pa/m^3^) in the three measurement regions is shown in Figure 8. The proportion between coarse particles (pa/m^3^) and bacterial concentrations (CFU/m^3^) in different altitudes (m) is presented in Figure 9.

The percentage of mesophilic bacteria within the coarse particle fraction was < 0.1%. The highest proportion of mesophilic bacteria (37 °C) was found in an altitude of ≤450 m with 0.14% as well as in the urban regions. The results show a decrease in coarse particles with increasing altitude. The lowest median value of 0.07% was found in the mountainous regions (Table 3).

## 4. Discussion

In this study, the total concentration of mesophilic bacteria and *Staphylococcus* sp. was investigated in the air of three different geographical regions to determine background concentrations which can be used as baseline values to assess bacterial emissions. Furthermore, the particle concentrations were measured to examine possible correlations between them and airborne mesophilic bacteria.

### 4.1. Concentrations of Total Mesophilic Bacteria

The lowest background concentrations of total mesophilic bacteria were obtained in mountain regions. The median values at urban and rural locations were two to three times higher than those in the mountains. The emission sources are predominantly natural and not anthropogenic in mountain regions; this could be a reason for the low concentrations. A comparison of cultivable airborne mesophilic bacterial concentrations in an altitude location on the Jungfraujoch at 3571 m above sea level was <10 CFU/m^3^ in mid-September, while in the urban area of Zürich-Irchel at 488 m were between 8.0 × 10^1^ and 1.2 × 10^2^ CFU/m^3^ [5]. Lee et al., 2019 conducted measurements of bioaerosols at sites in the mountains and at the seashore and used the data of an urban area as a reference. Their results showed lower values of bioaerosols at the seashore and urban sites than in the mountains sites [30]. The authors agree with Fröhlich-Nowoisky et al. (2016) who reported that trees and other living organisms in the mountains could produce high amounts of bioaerosols [31].

According to VDI 4253/Part 3, mesophilic bacterial concentrations are subject to seasonal and daily fluctuations [6]. Haas et al. (2013) determined the highest mesophilic bacterial concentrations (cultivated at 30 °C) of 2.5 × 10^3^ CFU/m^3^ in the winter months in the urban region [32]. The authors concluded that numerous mesophilic bacteria adhere to the high particle numbers in the city in winter. Miesebner (2014) determined mesophilic bacterial concentrations between 2.0 × 10^0^ and 6.4 × 10^1^ CFU/m^3^ in urban regions and between 1.0 × 10^0^ and 4.9 × 10^2^ CFU/m^3^ in rural regions in late autumn and winter months [33]. In Poland, a study recorded a mesophilic bacterial concentration of 6.5 × 10^1^ CFU/m^3^ in winter which was four times less than in spring [34]. The study by Kolk et al. (2009) showed that the lowest bacterial concentrations in the ambient air are between January and April and the highest in May [35]. For the period from July to October, they determined median values between 1.2 × 10^2^ and 2.3 × 10^2^ CFU/m^3^, which corresponds to the results of the present study in rural and urban regions for total culturable mesophilic bacteria at 30 °C.

In the present study the highest mesophilic bacterial concentrations were found at the urban measuring locations DB and GS, which is certainly due to the strongest emission sources (e.g., traffic, industry, people) in the environment. Rural locations such as AR, BB, JU-ST, KL and TS also had high mesophilic bacterial concentrations, which can be due to agriculture activities. Shaffer and Lighthart (1997) determined concentrations of 6.1 × 10^2^ CFU/m^3^ in an urban and 2.4 × 10^2^ CFU/m^3^ in a rural location [17]. The investigations by Reinthaler et al. (1999) measured median values of mesophilic bacteria in rural areas of 1.5 × 10^2^ CFU/m^3^ and in urban areas of 1.2 × 10^2^ CFU/m^3^ which are similar to the present study [36]. Haas et al. (2013) conducted airborne mesophilic bacteria measurements in an urban region over a period of one year and determined the same median value of 1.2 × 10^2^ CFU/m^3^ for the total mesophilic bacterial concentration cultivated at 30 °C [32]. In Beijing urban areas, Fang et al. (2008) recorded a median value of 1.4 × 10^3^ CFU/m^3^ for mesophilic bacterial concentration [37]. In the air of the larger cities, higher mesophilic bacterial concentrations can be expected.

### 4.2. Concentrations of Staphylococcus sp.

According to Fang et al. (2007), over 80% of detectable bacteria in air samples are Gram-positive [38]. In contrast to Gram-negative bacteria, Gram-positive bacteria have a thick cell wall which makes bacteria more resistant to external stresses, including heat, UV radiation and antibiotics. In this study, 11% of all mesophilic bacteria were found to be Gram-positive *Staphylococcus* sp. which were three times higher in urban and rural sites than in mountain regions. Lohberger (2016) obtained similar results for the background measurements with an impinger in rural areas and recorded a median value of 3.0 × 10^0^ CFU/m^3^ for the concentrations of *Staphylococcus* sp. [39]. Emission sources such as animal barns can contribute to bacterial pollution up to several kilometres away [40]. Ehgartner (2019) determined average values from 9.0 × 10^1^ CFU/m^3^ to 2.5 × 10^4^ CFU/m^3^ for the background concentrations of *Staphylococcus* sp. in the vicinity of animal stables by means of impaction [41]. However, it must be noted that airborne microorganism measurements carried out with different measuring devices can only be compared under defined conditions.

The occurrence of *S. aureus* in various environments has long been observed [42]. There is a high risk of contact with *S. aureus*, particularly in public places, not only in hospitals but also in the vicinity of sewage treatment plants or on farms with large numbers of animals [43]. *S. aureus* were not found in this study. Lohberger (2016) had the same results measuring background concentrations over a period of two years [39]. Gandara et al. (2006) determined a median value for outdoor concentrations of *S. aureus* of 1.3 × 10^1^ CFU/m^3^ [44]. Madsen et al. (2018) stated that the prevalence of *S. aureus* was very low in all seasons [45].

### 4.3. Concentrations of Fine and Coarse Particles

In the present study, the concentrations of particles smaller than 2.3 µm were approximately the same in the three investigated regions. Fine particles from 2.3 to 10 µm differed 1.3 times in their concentration in descending order: urban > rural > mountain. Haas et al. (2013) determined similar median values of 1.9 × 10^7^ pa/m^3^ for fine particles and 1.4 × 10^3^ pa/m^3^ for coarse particles in urban air [32]. However, urban locations had higher particle concentrations than rural and mountainous locations due to emissions from local anthropogenic sources, which can be exacerbated by meteorological conditions. Daily cyclic variations of dust particles, for example, are attributed to local emissions and prevailing meteorology [46]. Buchunde et al. (2019) detected a diurnal variation of fine and coarse particles with an increase in the morning and evening hours [47] most likely due to rush hour. In the study of Li et al. (2020) the highest mean of seasonal particle concentrations was found in the industrial zone with peaks during morning rush hour [48]. High levels of particulate matter, observed in large cities around the world, give cause for concern about their short and long-term effects on public health. Karagulian (2015) concluded in a review that in most regions studied around the world, natural sources account for a higher contribution in PM_10_ than in PM_2.5._ This indicated the influence of natural sources on coarse particles and anthropogenic sources on fine particles [49]. Menetrez et al. (2009) indicated that higher concentrations of microorganism were present in PM_10_ than in PM_2.5_ [50]. Both statements confirm the results of the present study, that the background concentrations of mesophilic bacteria correlate more with coarse than with fine particles.

### 4.4. Environmental Influencing Factors

The measuring locations of the present study were at different altitudes between 250 and 1900 m. The results show a negative correlation between mesophilic bacterial concentrations and altitude. The concentrations of fine particles were highest in areas between 450 m and 850 m whereas coarse particle concentrations decreased with increasing altitude. Fulton (1966) found that the concentrations of dust particles were an order of magnitude lower at 1600 m than at 690 m and even lower at 3127 m [51]. Mesophilic bacterial concentrations are not necessarily lower in mountain regions than in other areas. Lee et al. (2019) found higher mesophilic bacterial concentrations of 7.8 × 10^2^ CFU/m^3^ in a mountain region of Korea than in the coastal area [30]. Furthermore, in the Qinling Mountains in China, the concentration of airborne cultivable mesophilic bacteria was of 3.8 × 10^3^ ± 1.8 × 10^3^ CFU/m^3^ which was higher than those in the urban environment [52]. Southern European countries are often affected by transboundary air pollution from Saharan dust in summer which causes a reddening in the entire mountain range of the Alps and Apennines [53]. Bioaerosols and dust particles at higher altitudes may originate from a distant upwind source [54].

In this study, a negative correlation could be found between mesophilic bacterial concentrations and air humidity in the three geographical regions. Other investigations concluded that there are no correlations between mesophilic bacterial concentrations and air humidity [55,56,57,58]. Yao et al. (2010) noted that the relatively large size of airborne particles allows for absorbance of more moisture from the air when the humidity increases. This means the settling rate of particles also increases and as a result, there is a smaller concentration of mesophilic bacteria in the air [59]. In the present study, this could be a reason why there were fewer aerosol particles in the air in foggy weather. Gao et al. (2016) analysed the effect of haze on bioaerosols and determined a negative correlation [60].

In this study, however, highly significant differences (*p* < 0.001) were observed between mesophilic bacterial concentrations and temperatures. Gong et al. (2020), Harrison et al. (2005) and Di Giorgio et al. (1996) came to the same result that the concentration of mesophilic bacteria in the air increases with increasing temperature [14,57,58]. Haas et al. (2013) concluded that the total mesophilic bacterial concentrations decreased significantly with increasing ambient air temperatures whereas the air humidity has no influence on them [32]. Kolk et al. (2009) and Raisi et al. (2010) found no correlations between the two variables [35,56]. Other authors found correlations between concentrations of *Staphylococcus* sp. and temperature. Madsen et al. (2018) and Moon et al. (2014) reported that concentrations of these species tend to be temperature related [45,61]. As mentioned in the introduction, different air temperatures affect Gram-positive bacteria less than Gram-negative bacteria [62]. The increase of mesophilic bacterial concentrations with increasing temperatures are probably due to the positive correlation of coarse particles in the ambient air.

Regarding the weather conditions, it was observed in the present study that sunshine has no diminishing effect on mesophilic bacterial concentrations. It was found that the measured concentrations were as high on sunny days as on cloudy days. Lindemann and Upper (1985) explained that in sunny weather dried leaves become a strong source of bacterial aerosols [63]. The bacterial communities on leaves and in dust particles differ from each other by season. In the summer months, for example, plant associated bacteria dominate the air [64]. On the one hand, strong sunlight is considered harmful to mesophilic bacteria because they dry out faster in the air. On the other hand, the mesophilic bacteria can be ingested by raindrops. Polymenakou (2012) suggested that mesophilic bacteria might multiply in cloud water droplets via metabolic activities, because bacteria are able to survive harsh conditions such as extremely high or low temperature and strong acidity in liquid [65]. This fact may explain why Womack, et al. (2010) noted that the atmosphere is not the most extreme microbial habitat [66]. Sattler et al. (2001) could prove that bacteria grow actively in cloud droplets and therefore, cloud water should be considered as a microbial habitat [67]. This could be a reason why the mesophilic bacterial concentrations in this study were very high in cloudy weather conditions. This was also shown in the evaluations of fine and coarse particles in this study, which were equally high in both sunny and cloudy weather conditions.

Most mesophilic bacteria have an aerodynamic diameter between >1 µm and 10 µm and often adhere to coarse particles in the air. Adherence to dust particles may also increase the ability of viable bacteria to endure stresses such as ultraviolet rays, dry conditions, and low temperatures [68]. Low temperature is considered to be a limiting factor for cell activity in the air, although some studies have demonstrated that bacterial activity can occur at subzero temperatures. Besides temperature, the presence of oxidants and solar radiation presumably limit the survival of individual microbes in the air [69]. Jones and Harrision (2004) explained that pigmentation protects bacteria from solar radiation and the highest concentration of bacteria is found at rural sites [70].

If windy conditions prevail at the same time, it is quite conceivable that bacteria move from their natural habitat. However, it has been observed that there was no significant reduction on the measured concentrations. The concentrations of airborne mesophilic bacteria and *Staphylococcus* sp. did not appear to be influenced by wind conditions, as the results of the bioaerosol measurements showed in a Greek city [57]. Other authors reported that there are definitely positive correlations between mesophilic bacterial concentrations and wind speeds of 1–3 m/s [55,56,70]. Perez (2012) observed a positive correlation (rho = 0.34) between fine and coarse particles during days with and without Saharan dust [71].

At high wind speeds, deposition of coarse particles is more efficient, while the opposite is true for fine and ultrafine particles. To improve deposition, surrounding vegetation should have a large leaf area index but still be possible to penetrate. Air pollution passing over and not through the vegetation has no filter effect [72].

### 4.5. Correlations between Mesophilic Bacterial and Particle Concentrations

The comparison of mesophilic bacterial concentrations which were measured in parallel with dust particles in the present study showed only a slightly negative correlation between fine particles and the concentrations of mesophilc bacteria at 30 °C. As the fine particles in the air increase, the concentration of mesophilic bacteria decreases. The coarse particles showed a positive correlation to mesophilic bacterial concentrations. Haas et al. (2013) reported that the concentrations of airborne mesophilic bacteria have significant correlations with all particle fractions, especially with those of the coarse particles [32]. It was found that bacteria adhere to a large number of particles in the ambient air. Burrow et al. (2009) and Hara and Zhang (2012) observed a quantitative relationship between coarse particles and viable bacterial cells [54,73]. Shaffer and Lighthart (1997) and Zhao et al. (2010) found a correlation between airborne mesophilic bacteria and especially particles with aerodynamic diameters >3 µm [17,74]. Parat et al., 1999 achieved the same results [75]. Zheng et al. (2013) stated that particles with larger aerodynamic diameters and masses in the ambient air could carry more bacteria [76]. Naide et al. (2018) found correlations between fine dust particles and airborne microorganisms of sizes 5–10 µm, as well as between the concentrations of *S. aureus* and aerosol concentrations in particle sizes of 2–10 µm [77]. Polymenakou et al. (2008) detected large portions of bacteria at particle sizes of <3.3 μm as phylogenetic neighbours to human pathogens [78]. According to Madsen (2018), most *S. aureus* were associated with particles between 7 and 12 µm [45]. When there was low-anthropogenic pollution of the air, a positive significant correlation (*p* < 0.05) was found between bacterial protein and coarse particle fractions but not in fine fractions [79]. Coarse particles can move several miles away from their emission sources and fine particles even further [40].

The proportion of mesophilic bacteria within the dust particles in the air is an important issue, as it is assumed that microbial emissions can spread over large distances by adhering to particles. The present study indicates that the proportion of mesophilic bacteria and the coarse particle fraction were <0.1% and the highest proportion was found in the urban regions at an altitude of <450 m.

Obviously, the proportion of microorganisms in dust particles differs greatly from different emission sources.

## 5. Conclusions

This study provides background values for total airborne mesophilic bacteria, concentrations of *Staphylococcus* sp. and dust particles in unpolluted urban, rural and mountain regions in Styria, Austria for the summer period from July to October. The median concentration of cultivated mesophilic bacteria at 30 °C was 7.1 × 10^2^ CFU/m^3^ in mountain regions, 1.3 × 10^2^ CFU/m^3^ in rural regions and 2.1 × 10^2^ CFU/m^3^ in urban regions; whereas at 37 °C the median value was 2.3 × 10^1^ CFU/m^3^ in mountain regions, 6.9 × 10^1^ CFU/m^3^ in rural regions and 6.5 × 10^1^ CFU/m^3^ in urban regions. The median of *Staphylococcus* sp. was 3.0 × 10^0^ CFU/m^3^ in mountainous areas and 8.0 × 10^0^ CFU/m^3^ in urban and rural areas. The fine particle numbers (0.35–2.3 μm) were 1.2 × 10^7^ pa/m^3^ and the coarse particles (>2.3–10 μm) were 6.5 × 10^4^ pa/m^3^. Mesophilic bacteria had a positive correlation to coarse particles. These background values can be used as baseline values for the assessment of emissions in different geographical regions. To find out the interactions between mesophilic bacteria and particles in the air of different geographical regions, further studies over a year are recommended.

## Figures and Tables

**Table 1:** Total concentrations of mesophilic bacteria and *Staphylococcus* sp. in relation to weather conditions, altitude and region.

Bacterial Concentrations in CFU/m^3^	Environmental Factors		*n*	Median	Q1	Q3	Minimum	Maximum
Total mesophilic bacteria(30 °C)	altitude	≤450 m	55	235	128	345	0	1910
		>450 m–≤850 m	35	113	70	190	35	733
		>850 m	35	55	18	155	5	920
	weather	little cloud cover	25	175	80	273	5	1050
		heavy clouds	39	128	55	228	5	523
		foggy	8	18	15	59	5	80
		sunny	53	175	78	303	0	1910
	wind	slight wind	85	153	68	258	5	1910
		strong wind	18	149	18	463	5	1180
		windless	22	133	83	275	0	895
	region	urban	45	208	125	285	35	1050
		rural	40	133	65	266	0	1910
		mountain	40	71	20	163	5	920
Total mesophilic bacteria (37 °C)	altitude	≤450 m	55	88	53	200	0	825
		>450 m–≤850 m	35	48	35	83	13	340
		>850 m	35	20	8	40	0	753
	weather	little cloud cover	25	58	40	93	3	753
		heavy clouds	39	45	20	83	3	250
		foggy	8	13	5	24	3	30
		sunny	53	68	35	185	0	825
	wind	slight wind	85	55	23	98	0	825
		strong wind	18	20	10	173	3	563
		windless	22	75	43	125	0	298
	region	urban	45	65	48	193	13	340
		rural	40	69	31	134	0	825
		mountain	40	23	9	58	0	753
*Staphylococcus* sp.	altitude	≤450 m	55	8	3	18	0	75
		>450 m–≤850 m	35	8	5	10	0	55
		> 850 m	35	3	0	8	0	93
	weather	little cloud cover	25	8	3	10	0	93
		heavy clouds	39	5	3	8	0	75
		foggy	8	0	0	0	0	5
		sunny	53	8	3	15	0	55
	wind	slight wind	85	8	3	10	0	93
		strong wind	18	4	0	13	0	38
		windless	22	5	0	10	0	75
	region	urban	45	8	5	10	0	75
		rural	40	8	3	18	0	55
		mountain	40	3	0	8	0	93

*n* = number of samples; Q1 = first quartile and Q3 = third quartiles.

**Table 2:** Numbers of fine and coarse particles in relation to weather, altitude and region.

Particle Concentrations (pa/m^3^)	Environmental Factors		*n*	Median	Q1	Q3	Minimum	Maximum
Fine particles	altitude	≤450 m	55	1.06 × 10^7^	6.58 × 10^6^	2.03 × 10^7^	7.98 × 10^5^	8.02 × 10^7^
		>450 m–≤850 m	35	1.97 × 10^7^	1.02 × 10^7^	2.36 × 10^7^	4.42 × 10^6^	5.70 × 10^7^
		>850 m	34	1.11 × 10^7^	7.55 × 10^6^	2.33 × 10^7^	8.65 × 10^5^	5.57 × 10^7^
	weather	little cloud cover	25	1.06 × 10^7^	6.35 × 10^6^	1.48 × 10^7^	7.98 × 10^5^	5.60 × 10^7^
		heavy clouds	39	1.37 × 10^7^	7.11 × 10^6^	2.69 × 10^7^	3.18 × 10^6^	8.02 × 10^7^
		foggy	7					
		sunny	53	1.40 × 10^7^	8.01 × 10^6^	2.35 × 10^7^	8.65 × 10^5^	5.70 × 10^7^
	wind	slight wind	84	1.15 × 10^7^	7.60 × 10^6^	2.13 × 10^7^	8.65 × 10^5^	8.02 × 10^7^
		strong wind	18	1.07 × 10^7^	6.56 × 10^6^	2.33 × 10^7^	3.18 × 10^6^	4.31 × 10^7^
		windless	22	2.14 × 10^7^	7.05 × 10^6^	3.07 × 10^7^	8.98 × 10^5^	4.68 × 10^7^
	region	urban	45	1.16 × 10^7^	7.19 × 10^6^	2.18 × 10^7^	3.77 × 10^6^	8.02 × 10^7^
		rural	40	1.43 × 10^7^	6.72 × 10^6^	2.94 × 10^7^	7.98 × 10^5^	5.57 × 10^7^
		mountain	39	1.11 × 10^7^	8.18 × 10^6^	2.11 × 10^7^	8.65 × 10^5^	4.68 × 10^7^
Coarse particles	altitude	≤450 m	54	8.58 × 10^4^	5.27 × 10^4^	1,04 × 10^5^	2.75 × 10^4^	3.19 × 10^5^
		>450 m–≤850 m	35	7.38 × 10^4^	5.62 × 10^4^	1.05 × 10^5^	3.82 × 10^4^	3.30 × 10^5^
		>850 m	26	3.65 × 10^4^	2.48 × 10^4^	4.74 × 10^4^	5.48 × 10^3^	1.24 × 10^5^
	weather	little cloud cover	25	6.80 × 10^4^	3.96 × 10^4^	1.01 × 10^5^	2.07 × 10^4^	3.19 × 10^5^
		heavy clouds	37	6.57 × 10^4^	4.40 × 10^4^	9.84 × 10^4^	9.22 × 10^3^	2.32 × 10^5^
		foggy	9					
		sunny	53	6.98 × 10^4^	4.59 × 10^4^	9.74 × 10^4^	5.48 × 10^3^	3.30 × 10^5^
	wind	slight wind	80	6.07 × 10^4^	4.23 × 10^4^	9.52 × 10^4^	5.48 × 10^3^	3.30 × 10^5^
		strong wind	13	7.83 × 10^4^	5.55 × 10^4^	1.05 × 10^5^	9.22 × 10^3^	2.29 × 10^5^
		windless	22	8.66 × 10^4^	6.37 × 10^4^	1.12 × 10^5^	1.98 × 10^4^	2.16 × 10^5^
	region	urban	45	8.39 × 10^4^	5.96 × 10^4^	1.05 × 10^5^	2.97 × 10^4^	3.30 × 10^5^
		rural	40	6.34 × 10^4^	4.39 × 10^4^	9.94 × 10^4^	2.07 × 10^4^	3.19 × 10^5^
		mountain	30	4.64 × 10^4^	3.04 × 10^4^	7.83 × 10^4^	5.48 × 10^3^	1.99 × 10^5^

*n* = number of samples; Q1 = first quartile and Q3 = third quartiles.

**Table 3:** Proportion of coarse particles in relation to altitude and region.

Temperature of Cultivation	Environmental Factors		N	Median	Q1	Q3	Minimum	Maximum
30 °C	altitude	≤450 m	54	0.26	0.16	0.49	0.00	2.47
		>450 m–≤850 m	35	0.13	0.09	0.26	0.06	0.87
		>850 m	26	0.25	0.08	0.69	0.01	5.67
	region	urban	45	0.24	0.13	0.43	0.07	1.32
		rural	40	0.17	0.10	0.34	0.00	2.47
		mountain	30	0.24	0.08	0.46	0.01	5.67
37 °C	altitude	≤450 m	54	0.14	0.08	0.20	0.00	0.62
		>450 m–≤850 m	35	0.06	0.05	0.11	0.01	0.63
		>850 m	26	0.07	0.03	0.15	0.00	2.08
	region	urban	45	0.10	0.06	0.16	0.01	0.62
		rural	40	0.08	0.05	0.19	0.00	0.63
		mountain	30	0.07	0.03	0.15	0.00	2.08

N = number of samples; Q1 = first quartile and Q3 = third quartiles.

## Supplementary Materials

The following are available online at https://www.mdpi.com/1660-4601/17/24/9572/s1, Table S1: Median values of the CFU/m^3^ for the total mesophilic bacterial and *Staphylococcus* sp. concentrations (30 °C and 37 °C) of the measurement locations in the mountain, rural and urban regions.

## Abbreviations

Legend: The abbreviations of the measuring locations of Figure 1.

AF	Aflenz
AR	Arnfels
BB	Bockberg
BM	Bruck an der Mur
DB	Don Bosco
DL	Deutschlandsberg
GB	Gröbming
GL	Grundlsee
GR	Grebenzen
GS	Graz Süd
HG	Hochgößnitz
HW	Hochwurzen
JU	Judenburg
JU-ST	Judendorf-Straßengel
KA	Kapfenberg
KL	Klöch
KÖ	Köflach
KR	Krottendorf
LB	Leibnitz
LI	Liezen
PB	Plabutsch
RE	Rennfeld
SC	Schöckl
TA	Teichalm
TS	Thalersee
