# Supplementary material for: Background Concentrations of Cultivable, Mesophilic Bacteria and Dust Particles in the Air in Urban, Rural and Mountain Regions
Source: Int J Environ Res Public Health. 2020 Dec 21;17(24):9572. doi: 10.3390/ijerph17249572 (PMC7767401; doi:10.3390/ijerph17249572)
Supplement: Supplementary file 1 [file ijerph-17-09572-s001.pdf]

Supplementary Material

Table S1: Median values of the CFU/m^3 for the total mesophilic bacterial and *Staphylococcus* sp. concentrations (30° C and 37° C) of the measurement locations in the mountain, rural and urban regions

Region	Total bacteria concentrations CFU/m^3 (Median)		*Staphylococcus* sp. CFU/m^3 (Median)
**Mountain (n=8)**	**30 °C**	**37 °C**	**37 °C**
Teichalm	$1.8x10^1$	$2.0x10^1$	$5.0x10^0$
Grebenzen	$2.3x10^1$	$5.0x10^0$	$0.0x10^0$
Hochwurzen	$3.0x10^1$	$1.0x10^1$	$0.0x10^0$
Rennfeld	$3.8x10^1$	$2.0x10^1$	$0.0x10^0$
Arnfels	$1.6x10^2$	$1.8x10^1$	$0.0x10^0$
Plabutsch	$7.3x10^1$	$6.5x10^1$	$5.0x10^0$
Schöckl	$8.0x10^1$	$2.3x10^1$	$0.0x10^0$
Hochgößnitz	$1.7x10^2$	$6.8x10^1$	$5.0x10^0$
**Rural (n=8)**	**30 °C**	**37 °C**	**37° C**
Grundlsee	$4.5x10^1$	$1.5x10^1$	$2.5x10^0$
Gröbming	$7.0x10^1$	$4.0x10^1$	$5.0x10^0$
Krottendorf	$7.0x10^1$	$1.4x10^2$	$2.5x10^0$
Aflenz	$8.0x10^1$	$3.5x10^1$	$7.5x10^0$
Judendorf	$1.4x10^2$	$5.3x10^1$	$5.0x10^0$
Thalersee	$1.7x10^2$	$7.0x10^1$	$2.5x10^0$
Bockberg	$2.4x10^2$	$1.2x10^2$	$1.8x10^1$
Klöch	$4.6x10^2$	$1.4x10^2$	$1.8x10^1$
**Urban (n=9)**	**30 °C**	**37 °C**	**37° C**
Kapfenberg	$1.2x10^2$	$4.5x10^1$	$5.0x10^0$
Liezen	$1.5x10^2$	$3.5x10^1$	$1.0x10^1$
Köflach	$1.8x10^2$	$5.3x10^1$	$2.5x10^0$
Bruck an der Mur	$1.8x10^2$	$6.3x10^1$	$7.5x10^0$
Deutschlandsberg	$2.4x10^2$	$5.5x10^1$	$2.5x10^0$
Judenburg	$2.4x10^2$	$5.8x10^1$	$5.0x10^0$
Leibnitz	$2.6x10^2$	$7.3x10^1$	$1.0x10^1$
Don Bosco	$2.9x10^2$	$2.2x10^2$	$1.0x10^1$
Graz Süd	$4.2x10^2$	$2.0x10^2$	$1.5x10^1$
